# A mobile health application for patients eligible for statin therapy: app development and qualitative feedback on design and usability

**DOI:** 10.1186/s12911-023-02221-4

**Published:** 2023-07-19

**Authors:** Weidan Cao, Lang Li, Puneet Mathur, John Thompson, M. Wesley Milks

**Affiliations:** 1 Department of Biomedical Informatics, The Ohio State University, Columbus, OH 43210 USA; 2 Department of Research Information Technology, College of Medicine, The Ohio State University, Columbus, OH USA; 3 Division of Cardiovascular Medicine, The Ohio State University College of Medicine, Columbus, OH USA

**Keywords:** Statin intolerance, Mobile health application, Symptom logging, Doctor-patient communication, Disease co-management

## Abstract

**Background:**

Cardiovascular disease is the leading cause of death in the United States (US). Despite the well-recognized efficacy of statins, statin discontinuation rates remain high. Statin intolerance is a major cause of statin discontinuation. To accurately diagnose statin intolerance, healthcare professionals must distinguish between statin-associated and non-statin-associated muscle symptoms, because many muscle symptoms can be unrelated to statin therapy. Patients’ feedback on muscle-related symptoms would help providers make decisions about statin treatment. Given the potential benefits and feasibility of existing apps for cardiovascular disease (CVD) management and the unmet need for an app specifically addressing statin intolerance management, the objectives of the study were 1) to describe the developmental process of a novel app designed for patients who are eligible for statin therapy to lower the risk of CVD; 2) to explore healthcare providers’ feedback of the app; and 3) to explore patients’ app usage experience.

**Methods:**

The app was developed by an interdisciplinary team. Healthcare provider participants and patient participants were recruited in the study. Providers were interviewed to provide their feedback about the app based on screenshots of the app. Patients were interviewed after a 30 days of app usage.

**Results:**

The basic features of the app included symptom logging, vitals tracking, patient education, and push notifications. Overall, both parties provided positive feedback about the app. Areas to be improved mentioned by both parties included: the pain question asked in symptom tracking and the patient education section. Both parties agreed that it was essential to add the trend report of the logged symptoms.

**Conclusions:**

The results indicated that providers were willing to use patient-reported data for disease management and perceived that the app had the potential to facilitate doctor-patient communication. Results also indicated that user engagement is the key to the success of app efficacy. To promote app engagement, app features should be tailored to individual patient’s needs and goals. In the future, after it is upgraded, we plan to test the app usability and feasibility among a more diverse sample.

**Supplementary Information:**

The online version contains supplementary material available at 10.1186/s12911-023-02221-4.

## Introduction

### Statin intolerance and statin discontinuation

Cardiovascular disease is the leading cause of death among men and women in the United States (US). Statin (HMG-CoA reductase inhibitors) agents are the most effective and widely used medications to lower cholesterol and reduce the risk of atherosclerotic cardiovascular disease (ASCVD) [1–3]. A meta-analysis including 76 randomized controlled trials involving 170,255 participants found that statins are highly efficacious (i.e., a 20% risk reduction in cardiovascular disease (CVD) death and a 10% reduction in all-cause mortality) [4]. Despite the well-recognized efficacy of statins, therapeutic adherence rates among US adults are low, and statin discontinuation rates remain high [5–8]. One study found that 55.1% of patients were nonadherent and 44.7% of patients discontinued statin treatment during a one-year follow-up period [9]. Statin-associated adverse effects that have been described include muscle pain or weakness, cognitive dysfunction, and gastrointestinal issues, any of which can lead to statin intolerance, which is defined as “the inability to continue the use of a statin in a dosage sufficient to reduce individual cardiovascular risk due to the development of symptoms and/or laboratory abnormalities that coincide with the initiation or dose escalation of a statin” [10].

### Premature statin discontinuation

Statin intolerance is a major cause of statin discontinuation [6, 10–15] and can be further divided into complete intolerance (i.e., the inability to tolerate any dose of any statin) and partial, or “titrational,” intolerance (i.e., the inability to tolerate some doses of some statins) [16]. As many as 20% of patients experience partial statin intolerance [6], whereas according to one source, fewer than 5% of patients experience complete statin intolerance requiring statin discontinuation [6, 11, 15]. Most patients who experience partial intolerance can still use statins with the titrational guidance of their healthcare providers; however, many patients abandon medication use independent of medical advice. Premature discontinuation leaves patients at risk for first time and recurrent cardiovascular events.

### The need to facilitate distinguishing between perceived and real statin intolerance

To accurately diagnose statin intolerance, healthcare professionals must distinguish statin-associated from non-statin-associated muscle symptoms because many muscle symptoms can be unrelated to statin therapy [17]. Muscle symptoms are commonly reported by middle-aged or older patients who are not on statin treatment [18], and exacerbations of such symptoms can be due to increased intensity, prolonged duration, or new forms of physical activity, particularly if from a sedentary state. In addition, there are no adequately sensitive and specific objective biomarkers for the diagnosis of statin intolerance, given that the creatine kinase (CK) level may rise and fall independent of symptoms, and individuals may experience limiting symptoms in the absence of CK elevations. Once diagnosed, there is no consensus for management of statin intolerance [6, 16]. Moreover, the nocebo effect (i.e., patients’ perception and/or subjective experience of a side effect as a result of the expectation of harm from a drug), a well-established phenomenon that has gradually gained attention in cardiovascular medicine [18] further complicates statin intolerance diagnosis [19, 20]. Therefore, there is a great need for studies focusing on strategies and/or tools to help physicians manage patients with statin intolerance.

### Potential of mobile health apps

Mobile health (mHealth), defined as “medical and public health practice supported by mobile devices, such as mobile phones, patient monitoring devices, personal digital assistants (PDAs), and other wireless devices,” [21] and mobile applications (apps) for health purposes have gained increasing significance in health care, especially in chronic disease (particularly CVD) management [22]. The most widely used features of mHealth interventions for chronic disease management include system assessment, reminders, and tailored feedback to app users [22]. The most effective features of apps for self-management of CVD have included healthy behavior tracking, self-monitoring, patient education, and tailoring [23]. Mobile apps are highly valuable tools to educate patients, facilitate physician–patient communication, and streamline patient care [24]; apps are also effective in helping patients with CVD improve clinical outcomes [25].

### Study objectives

To the best of the authors’ knowledge, patients using existing apps that target improvement in CVD outcomes are used for disease self-management in general, whereas no apps have yet been developed to manage statin intolerance specifically. Given the potential benefits and feasibility of existing apps for CVD management and the unmet need for an app specifically addressing statin intolerance management, we sought to develop an app for this targeted purpose.

The are several reasons why we decided to develop a new app. First, this pilot usability study has the potential to inform development of future iterations of an app that could be freestanding, as it is currently, or integrated into a broader electronic health record (EHR) system. Usability, feasibility, and efficacy of future iterations of the app would be tested with the potential for EHR integration. In addition, apps can send out push notifications to patients’ smartphones to remind them to log symptoms and push notifications can also increase user engagement. Moreover, it is possible to incorporate patient-reported data via apps into the EHR [26], and doing so would streamline the potential for 2-way communication between the patient or user and provider with reference to data collected, both in the domain of muscle symptoms and the domain of biometric data such as vital signs and physical activity (e.g., steps taken). Second, existing CVD management apps are general, and specifically designed novel app was felt to have increased potential to better tailor to users’ needs [27]. For instance, the patient education section can present very specific information on statin medication and the importance of statin adherence.

The app was designed based on self-determination theory [28] to motivate app users to engage in healthy behaviors [29]. Self-determination theory posits that if the contextual condition satisfies basic psychological needs, such as autonomy (i.e., the feeling of one’s willingness and volition to achieve one’s goals or perform a behavior), competence (i.e., the self-efficacy to achieve the goals or to perform the activities), and relatedness (i.e., the feeling of being connected with and supported by others), people are more likely to be internally motivated to initiate health behavior, fulfill defined goals, and/or positively change their behavior [28].

Studies found that certain app features or functions that help satisfy users needs of autonomy, competence, and relatedness may work to motivate users to fulfill their desired goals [29] and to engage in behavior change [30]. For instance, reminders and goal-setting features will help support the need of autonomy. Symptom logging or self-monitoring will help support the need of competence. Messaging and news feeds will help support the need of relatedness [29].

In addition to describing the design process of the app, we also planned to collect both patients’ and healthcare providers’ feedback about the app because the ultimate purpose of the app is to facilitate the provider-patient relationship for co-management of the risk for CVD. The objectives of this study are as follows:

1. to describe the developmental process of a novel app designed for patients who are eligible for statin therapy for the purpose of lowering the risk of CVD,
2. to explore healthcare providers’ feedback of the app, and
3. to explore patients’ app usage experience.

## Methods

### Ethical consideration

This project was approved by the institutional review board (IRB) of The Ohio State University (OSU, protocol number: 2019H0368). This project was also evaluated and monitored by the risk assessment team of Information Technology (IT) Risk and Compliance at OSU Wexner Medical Center. Participant privacy and data confidentiality and security were ensured. Participants were provided written or digital informed consent and their participation was voluntary. Any change of the protocol or study procedure was approved by the OSU IRB and the risk assessment team.

### App development process

**Fig. 1:**
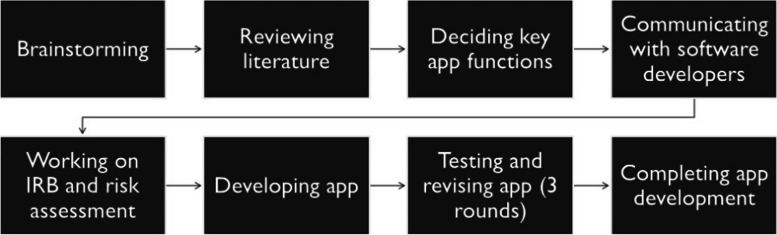
App development process

The app was developed by an interdisciplinary team, with members from cardiovascular medicine, bioinformatics, communication, and IT. The interdisciplinary team met regularly to decide the goals and the key features of the app. The team also systematically reviewed literature on the features and effects of mobile health apps, app development and deployment, mHealth-app involved interventions focusing on heart health. The key functions of the app were decided based on cardiologists’ clinical experience, potential user needs, and healthcare provider needs obtained from existing literature. The research team, then, communicated their ideas with the software developers to see the feasibility of implementation. During the app development process, the IT experts communicated with the rest of the team regularly to ask questions and to update the process of app development. A beta version of the app was created and three rounds of testing and revision were conducted among the members of the research team. Figure 1 displays the app development process.

### Study design

One of the aims of the study was to collect the feedback about the app from healthcare professionals and patient users; therefore, qualitative methodology (e.g., interviews) was used to fulfill the purpose. Semi-structured interviews were selected to collect in-depth feedback on app features, app usability, app usefulness, as well as app use facilitators and barriers. For healthcare providers, they were provided with screenshots of the app during an online interview. For patient users, they were invited to use the app for 30 days and then provide their feedback about their app use experience during an online interview.

### Inclusion and exclusion criteria

#### Healthcare provider participants

The healthcare professionals (e.g., doctors, nurses, pharmacists) who specialize in cardiovascular diseases and currently work at the Ohio State University Wexner Medical Center (OSUWMC) were eligible to participate in the study. Healthcare professionals who do not directly interact with patients were excluded.

#### Patient participants

One of the authors’ (MWM, a specialist in preventive cardiology) patients eligible for statin therapy, seen in the 12 months prior to enrollment, were recruited. The eligibility criteria of the participants were as follows (every item necessary for inclusion): age 18 years or older, with an indication for statin therapy based on contemporary guidelines, own and use an iPhone regularly, potential current or prior statin (partial or complete) intolerance (defined as: use of ≥ 2 statin-dose combinations in the past, or having a diagnosis of statin intolerance (ICD-10 Z78.9)). The exclusion criteria were: patients who were pregnant or nursing or patients who had serious reactions to statins. No participant was excluded from the study based on race or ethnicity.

### Recruitment

#### Healthcare providers

One author (MWM), a physician specializing in preventive cardiology at OSUWMC, contacted eligible healthcare professionals for the study. The recruitment took place in an online private setting. The research team then emailed or called the potential provider participants to gauge interest in participation. For those who were interested in participating, the research team then scheduled an online interview and obtained their verbal consent at the beginning of the online interview using online meeting tools such as Microsoft Teams.

#### Patients

Recruitment phone calls and emails were made to contact potential qualified participants by a research staff. If needed, a research staff member would answer the questions the potential participants might have. Interested participants received a link to fill out a short REDCap [31, 32] survey and then started to use the app at a later time.

### Procedures

#### Healthcare providers

The semi-structured interview included open-ended questions such as the participant’s role in health care and their views on the features of the app. The interviews took approximately 25–30 min, were conducted in a private setting online using an online meeting tool (e.g., Microsoft Teams), and underwent audio- or video-recording using the online meeting tool. Participation was voluntary and the provider participants did not receive any incentives.

#### Patients

Participants used the app for an intended 4 weeks. If needed, a member of the research team functioning as a technology navigator helped the participants install the app on their personal iPhone and answer their questions related to the app or the study, through emails or follow-up phone calls. During the 4 weeks, they were notified by the app push notifications to log the muscle-related symptoms every 5 days. A health tip was sent along with the push notifications. The participants were expected to log the symptoms as required and to read the heart health information provided on the app. Blood pressure, heart rate, body weight, and step counting were expected to be entered by participants manually to the app or were synched to the app from Apple Health Kit. After 30 days of app usage, participants participated in an online interview. The online interviews were audio-recorded or video-recorded depending on participants’ preferences using an online meeting platform (e.g., Microsoft Teams) and transcribed by the online meeting tool. A gift card in the value of $50 was offered to participants through emails. The interview was about the app users’ experience of the app, such as whether the app was easy to use, the other features to be added, etc.

### Data analysis

Recordings of video or audio interviews were transcribed and coded. Data were de-identified prior to data analysis to ensure confidentiality. Each participant was assigned a unique ID. Thematic framework analysis was employed to extract themes. The analysis process (involving steps such as familiarizing with data, generating codes, exploring themes) that has been employed in previous studies [33–35] was used.

## Results

### Statinterface design

**Fig. 2:**
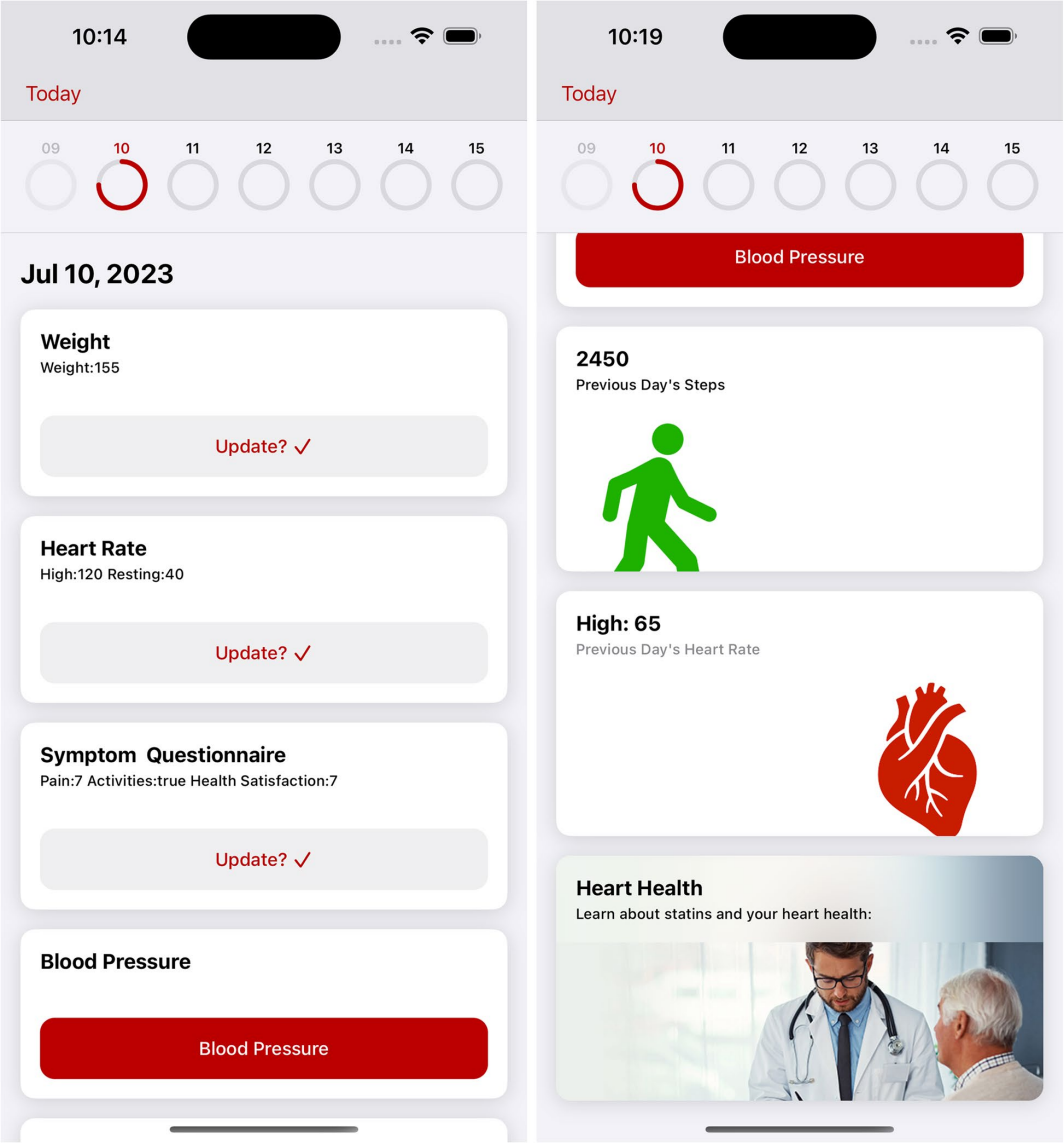
Screenshots presenting major features of the app

The app, named Statinterface, was developed and the basic features of the app included symptom logging, blood pressure, heart rate, weight, step tracking, patient education, and push notifications to send health tips and remind users to log their symptoms. This app does not provide direct CVD risk estimation to patients using the app, but patients targeted for app use were at a CVD risk level eligible for modification by statin therapy based on the inclusion criteria. Please see Fig. 2 with screenshots presenting the major features of the app. We have the permission to use all the images included in the app as they are stock images purchased via university contract with istock.com.

**Fig. 3:**
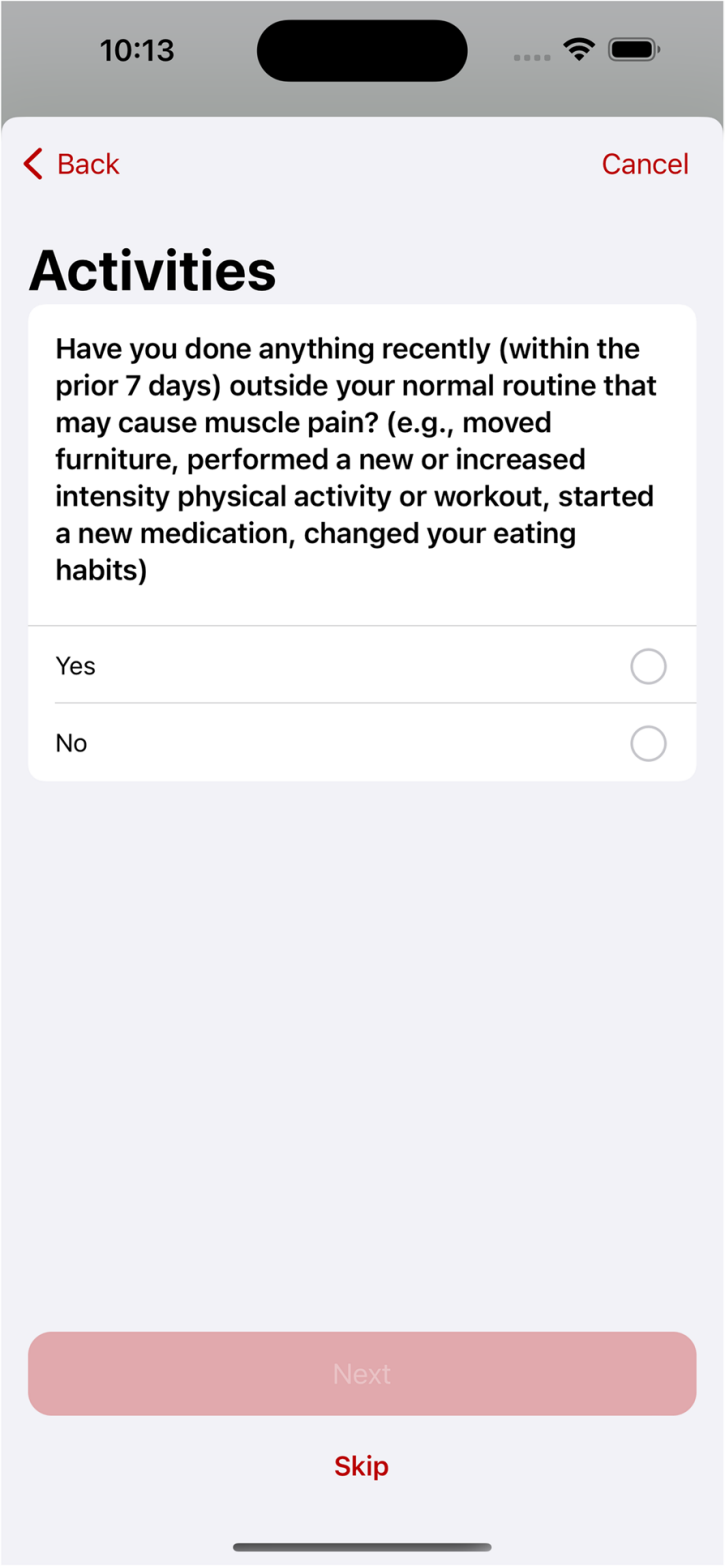
Sample screenshot of symptom logging

Regarding the symptom logging feature, patients could log any potential muscle-related symptoms on a daily basis. The frequency of symptom logging could be decided based on user’ needs and/or on their health provider recommendations. An example of the questions for symptom logging is: “Have you done anything recently (within the prior 7 days) outside your normal routine that may cause muscle pain (e.g., moved furniture, performed a new or increased intensity physical activity or workout, started a new medication, changed your eating habits)?” A sample screenshot of symptom logging is shown in Fig. 3. The questions asked in symptom logging were designed based on one source [16] and were presented in Appendix 1. This feature can help healthcare providers distinguish muscle-related symptoms of statin intolerance from other causes of muscle pain, although distinction between them is highly challenging and may be subjective in many individuals.

Patients could also manually enter their systolic and diastolic blood pressure, weight, high and resting heart rates. Daily steps and heart rate recorded by iPhone could also be synched to the app.

**Fig. 4:**
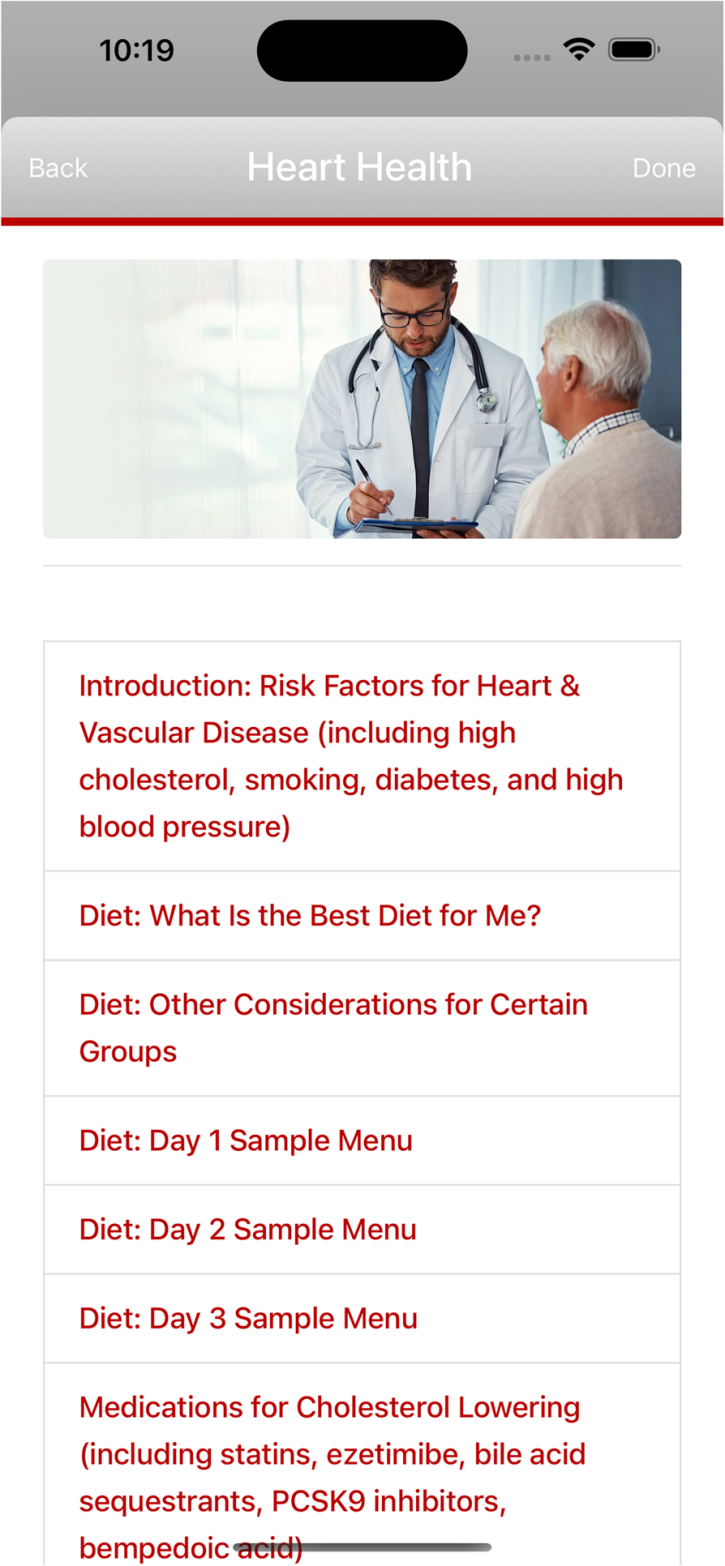
Sample screenshot of patient education

The patient education feature included introduction of risk factors for heart and vascular disease, diet recommendations, introduction of medications and supplements that can promote cholesterol or triglyceride lowering. A sample screenshot of patient education are shown in Fig. 4.

The purposes of the push notifications are 1) to remind users to log their muscle-related symptoms, and 2) to send health tips to users to promote a healthier lifestyle. An example of a push notification with a health tip is: “Fill out symptom questionnaire today. Health Tip: Try sauteeing or roasting (with a low-saturated-fat plant oil and minimal added salt) a different vegetable this week and giving it more-than-usual “real estate” on your dinner plate.”

### Healthcare providers’ feedback on app acceptability and sustainability

We used Proctor’s [36] framework to group the results of both providers’ and patients' app feedback. The five healthcare providers interviewed provided valuable feedback based on the screenshots of the app. The healthcare providers were all pharmacists associated with the medical center of The Ohio State University.

#### General app acceptability

All the providers provided positive overall feedback towards the app. For instance, providers mentioned:

“I think on the whole I like it.” (H5).

Some providers provided positive comments on the specific features of the app, such as sample menus presented in the patient education section and push notifications.

“I really like the features that are on it so far. The presentation is really nice, especially I like the sample menus.” (H4)

“I really like the push notifications. I think those are really helpful and I like that you have a kind of health tip on there. I love when things have diet plans I saw there. If you like different diet plans 'cause I think patients really like having examples so you can say like eat less saturated fats, eat more fruits and vegetables but when you show him like what a day could look like, I feel like that's really helpful. So I like that that that's incorporated.” (H1)

Although the providers were generally quite positive about the functions and the potential benefits of the app, much focus was also given to how the app could be improved. Suggested areas that could be improved included symptom tracking, blood pressure logging, patient education, steps tracking, and other important features to be added to the app.

#### Symptom tracking acceptability

Three out of 5 providers mentioned that the question to track pain level in the symptom tracking section was somewhat general. It asked the users to track general pain that could include all types of pain, such as ear pain or breast cancer pain that is not related to muscle or to the side effects of statins. If pain is tracked in a general way, this question is less important in that it is hard for health providers and patients to decide whether or not the pain is due to the side effects of the medication.

“Yes, we've lot of patients that have like other types of pain as well and that could just it does get kind of confusing when we're evaluating for statin intolerance.” (H4)

Providers were more interested in newly started muscle-related pain and would like more specific information about the pain.

“You know, scale is for that. I specifically would want to know if for the new pain could potentially be statin related.” (H4)

“Well, I guess I'm thinking like if somebody, I guess what we run into like people will be like well, I have pain from I've low back pain and I have low back pain for the last 20 years. But I have weakness in my lower extremities for the last week since I started my statin. I just worry that the answer you're going to get is I've had this pain for 20 years when really what you're trying to get, if I is, I've had these symptoms for the last week and, and maybe that's not I mean maybe.” (H5)

#### Tracking steps acceptability

Two providers mentioned that it was beneficial to have the feature to log the users’ daily steps. However, only measuring the steps may not be enough since they do not indicate the intensity and duration of the exercise.

“Even having the option to track like you know, the number of moderate intensity minutes in a day or high intensity minutes in the day.” (H5)

“But that may be helpful in a way, and I wonder if there's like if there's a way to translate that more so into duration and intensity …” (H3)

#### Blood pressure tracking acceptability

Some healthcare providers noted that the terms “systolic” and “diastolic” in the blood pressure tracking app section needs some explanation in the app, based on their concern on some patients’ levels of health literacy. If simple explanations are provided to explain the two terms in the app, patients are more likely to document correct information that will be useful for disease management.

“… especially like I, I noticed in the questionnaire, one of the…, maybe it wasn't tracking one of the things was systolic blood pressure. They may not know what systolic blood pressure is.” (H2)

“Like including somewhere like the systolic is the top number, diastolic is the bottom number” (H5)

#### Patient education acceptability

While the providers agreed that the patient education could be a valuable feature, some expressed concern that patient might start a medication or a supplement introduced in this section without first consulting their healthcare provider. Thus, they thought it is necessary to include a note (e.g., talk to your doctor before any action) to patients.

“So in general that what you have in that box as far as the information that would be offered looks propriate, you could even just, I don't know, make it seem something like oh, triglyceride goals are very patient specific. Make sure to talk to your doctor before starting any, you know, medications for lowering your triglycerides here or something patients that you might be on if you had high triglycerides and then listing out the, the medications as you have them.” (H4)

#### App appropriateness and feasibility

Some provider commented on appropriateness and feasibility of the app, how it is considered relevant and feasible for everyday use, and pointed out the potential benefits of facilitating provider-patient communication.

“I think this will be a way to you. You can tell me what's going on day-to-day between our visits and it'll help us at our with our conversations, even if it's something like that.” (H3)

#### App sustainability

Some providers had concerns that some users might be less motivated to use the app, especially for an extended period of time.

“I don't know that the patient will find much benefit in this unless they're having these symptoms and they really want to track it, but I don't know what the patient motivation level will be to persist with this long term.” (H3)

They agreed that one way of motivating the users could be to let the users have a clear understanding of the purpose and the potential benefits of the app. The purpose and benefits can be communicated in the app or by a provider in a face-to-face setting and the healthcare provider can be the advocate of the app.

"Yeah, and I think even if the benefit of the provider can say you know this will really help us like navigate this together, or saying like I know you have trouble with medications in the past.” (H3)

“I would hope by whatever healthcare provider is advocating for this app for their use.” (H2)

#### The need to add trend/summary report

In addition to providing some feedback about the existing features of the app, some providers mentioned the possibility of adding more crucial app features to better engage users. For instance, 3 out of 5 providers mentioned that it is necessary to include a trend or summary report of the tracked symptoms, so both patients and providers can use as a reference for treatment decision making and disease management.

“I do think that that would be great to be able to trend it up and see if there's a pattern to the symptoms that they're having.” (H5)

“So even if it's not immediately captured but just to say, you know, this is where you're yeah, if they can see that trend of their pain scales of versus when they stopped the medicine or held it or skipped it.” (H3)

#### The need to add contact doctor feature

Several (2 out of 5) indicated the need to include a feature for patients to contact their doctors especially during urgent situations. Alternatively, it would be better to let them know that the information they enter is not monitored by a healthcare provider and make it clear to users in the app on when to contact their healthcare provider.

“Yeah, I think one challenge is that, you know, let's say a patient enters a heart rate of 150 and there is no like escalation to provider. I think we would just have to make it clear. Or that there is no response immediately to that this wasn't monitored.” (H2)

### Patients’ 30-day app use feedback on app acceptability and sustainability

#### General app acceptability

All the 6 participants downloaded the app and used it for 30 days. The patient participants’ age ranged from 27 to 67, with a mean age of 55.83. The participants, all white, included two males and four females. After the app usage, they expressed their app usage experience during the online interview. All of them indicated that the app was easy to install and easy to use. For instance, one user said:

“Install process was, you know, fairly easy and straightforward.” (P3).

When it comes to the most important feature of the app, 4 out of 6 participants mentioned symptom tracking.

“Then I notice I have most of my pain on my right side, which I didn't pay attention before, but now I can pinpoint more.” (P5)

Two out of 6 users indicated that the patient education was helpful and 4 out of 6 users said the app helped improve their dietary choices. 3 out of 6 users indicated that the app acted as a motivator and helped improve their physical activities.

#### Symptom tracking acceptability

The users also mentioned some areas of the app that they found confusing about. Four out of 6 users pointed out the question about pain level in the section of symptom tracking was somewhat general and some did not feel comfortable using just a number to describe pain and indicated other ways to document the pain.

“Just I'm not comfortable saying I have a pain level of five” (P5)

“There was no place to mark where my pain was.” (P2)

Some users made great suggestions on how to improve this question, such as including a space for users to use text to describe the pain or to note the things to be discussed with the doctor during the next doctor’s appointment.

“What you're feeling or or a notation that would remind you that this is something that you feel like you really need to talk to your doctor about, or whatever. I think that would help.” (P2)

#### The need to add trend/summary report

In terms of symptom tracking, 4 out of 6 users thought it would be better if the app would have a feature of symptom trend or summary report, based on the symptoms they entered daily.

“I think it would be helpful if there was a way because right now there might be a way I'm just not aware of it. I can't see like any like chart or diagram or anything that like shows my collection of symptoms.” (P1)

“It's there once you track it, you could still have that there, but if you want to track it and really know the difference from one day to the next or week to the next week, to be able to write down.” (P2)

#### The need to let doctors access the patient-reported data

One user asked the interviewer if their physicians had access to their reported data using the app. Three out of 6 users mentioned that it would be better if their doctors could see their logged symptoms.

“So we're how, how did that physicians interact with it? Is the information actually going to go back into EPIC or do they have to use a different interface to look at?” (P3)

“If, if they had access, if the doctors had access to all this information that I have here that would be, absolutely wonderful.” (P4)

They believed that doctors’ access to the data will help motivate them to keep using the app and will help monitor emergent situations.

“I mean, … if somebody ends up having some kind of emergent issue through the app and there's not a high level of alerting or some way to then for physician follow up it it it typically starts to lack when the physicians side not on the adoption so.” (P3)

#### App tailoring

Some users mentioned the importance of app tailoring. Specifically, the symptom tracking and patient education, or health tips sent in push notifications need to be tailored.

“So you know it, depending on who you give it to, maybe tailor it a little more.” (P2)

This also echoed the issue that the pain question was somewhat general and some patients might enter the types of pain that were not related to muscle.

“For breast cancer survivor, however, I have severe pain under my breasts and that's where my pain is.” (P2)

Patient users also mentioned that patient education section needed to be tailored.

“You know, eat healthier oils or, you know, there was something that was so generic in high level about it. Like I don't think there was like a specific, you know, like information there.” (P3)

They also indicated that tailoring could be based on several user factors, such as users goals and/or needs. First thing to be considered is if the user needs patient education/health information or not. If yes, then, the second thing to be considered is what information that they need most based on their goals.

“You can even ask, I mean, do I need education about it? So, like, if if I'm trying to lose, if I'm trying to reduce my cholesterol and I need education, then I think having some information about the difference in the facts and the, you know, hydrogenated oils and all that other stuff could be really helpful, you know, to to come that way. If my goal is to lose weight is probably a different, you know it's a different set of information. So I think branching the information off of a set of goals, you know as you come in.” (P3)

“Uh, you know, if if anything were more tailored to specifically what somebody was trying to accomplish, I mean, because again, I think it can be handled up front where if somebody identified what their goal is, right, you know, it's my goal weight loss is my goal to try to reduce my cholesterol and you know, so I I think there's probably, you know, different goals that I would be trying to accomplish. And then if the information coming was more tailored to those.” (P3)

#### App appropriateness and feasibility

Patient participants also commented on the appropriateness and feasibility of the app. For instance, one expressed how the app can be incorporated into everyday life and acts like a motivator for physical activity:

“I know that this is like kind of simple and basic, but when I see like the little thing that's here's what your previous day's steps were. It is definitely a motivator to be like, oh, I should, you know, park farther away or, you know, do something so that I actually go walk around some more.” (P1)

#### App sustainability

Three out of 6 users planed to or did talk to their healthcare providers about the app and one (P6) of them mentioned that it would be helpful for doctor-patient communication in the long run.

Users sometimes were less motivated to log symptoms for an extended period of time, if the logged data would not be sent to their physicians. One user pointed out the issue of lack of motivation to continue using the app, due to the inconvenience of manual data entry, and due to the doubt of the value of the app.

“So I, I think any of those things that they're required that manually input just it, it either wasn't super convenient or the motivation wasn't there clear why to put it in.” (P3)

“'cause, everybody has so many apps on the phone. It's like, I'm not sure I saw enough value that I would continue to use it.” (P3)

“This information is going to go directly to my physician or clinician, you know, OSU and they're going to better help me get my medicines under control and kind of get rid of all these side effects. That's pretty good motivation for me to use it.” (P3)

In sum, providers and patients provided comprehensive feedback about the app acceptability and sustainability. A summary of the app features with issues and ways to improve the features was presented in Table 1.

**Table 1:** Providers’ and patients’ feedback about statinterface

	**Feature to be Improved**	**Details of the Feature**	**How to Improve**
**Providers' Feedback**	Symptom tracking	Questions related to muscle pain	Questions need to be more specific
	Steps tracking	Daily steps tracking	Tracking should also include exercise intensity and duration
	Blood pressure tracking	Daily tracking of systolic and diastolic blood pressure	Add notes to explain "systolic" and "diastolic" in easy-to-understand terms
	Patient education	The section has introductions on medication and supplements	Need to include a note to remind patients to ask doctors first before starting any medications or supplements introduced in this section
	Symptom trend report/summary	Not included in the app	Need to add the function
	Contact doctor	Not included in the app	Need to add the function
**Patients' Feedback**	Symptom tracking	Questions related to muscle pain	Need to provide ways to mark the pain or space to explain the pain in text
	Symptom trend report/summary	Not included in the app	Need to add the function
	Doctors' access or feedback to logged symptoms	Not included in the app	Need to add the function
	Patient education	Information is somewhat generic	Need to tailor the information

## Discussion

### Principle findings

In this study, we described the design process of the app, Statinterface, and provided results on physician feedback about the app, as well as results on the patients’ app usage experience. Both providers and patients provided comprehensive feedback for the app. Overall, both parties provided positive feedback about the app in that it was easy to use, and the features were helpful. In the interviews, both parties focused on the app features that could be improved and the important features that needed to be added in the future.

Areas to be improved mentioned by both parties included the pain question asked in symptom tracking and the patient education section. The pain question was somewhat general to users and no free text box was provided to document more details. As for the patient education section, patients mentioned that it was important to tailor app content and format to individual needs and goals, whereas physicians thought that adding a note to ask patients to consult their physicians before taking any actions after reading the patient education section was crucial. Both parties agreed that it was essential to add the trend report of the logged symptoms. Both parties agreed that some users might be less motivated to use the app, especially for an extended period of time. Some patients mentioned that physicians’ access to the logged data would make the app more useful and might increase app engagement. Therefore, based on the findings, we plan to update the app and then test its usability and feasibility.

### Implications and applicability of findings

The results of provider interview indicated that providers were willing to use patient-reported data for disease management and they perceived that the app had the potential to facilitate doctor-patient communication. This result is in agreement with a study including an interview of healthcare providers showing that providers recognized that patient symptom tracking could be useful in a variety of ways: facilitating disease diagnosis and management, enhancing provider-patient communication, and motivating and educating patients [37]. That study also indicated that healthcare providers lacked the time to review patient logged data [37]. These barriers could be overcome if the app generated a summary of the symptoms for healthcare provider review and a nurse care coordinator was assigned to review or monitor patient logged data. A trend or summary report would save review time. If a physician does not have much time to review the logged symptoms, a nurse could be designated as a care coordinator to lead the care and monitor the symptoms. Previous studies have documented the feasibility of employing a nurse care coordinator in managing patient-reported outcomes and found that benefits such as improved patient outcomes can be achieved as a result of designating a nurse care coordinator who monitors the disease status, and has in-person interactions with patients [38]. When necessary, the nurse can contact the physician for decision making.

Several options exist in terms of how healthcare providers can have access to the patient-reported data. One way is that patients can show their symptoms logged on their app to their healthcare providers during an office visit. Another way is to incorporate the logged symptoms to EHRs. A study has shown that incorporating patient-reported data into EHRs would facilitate patient-centered care and enhance patient health outcomes [39]. More studies can be done in the future to explore physicians’ attitudes and preferences about incorporating patient-reported data into EHRs.

Another important implication of the study is that user engagement is the key to the success of app efficacy [27]. To promote app engagement, app features should be tailored to individual patients’ needs and goals. This conclusion is in agreement with the findings of the systematic review that there is no one-size-fits-all app; tailoring is needed [40]. In addition to considering patients’ goals and needs, other patient factors can be considered when tailoring the app to users, such as disease characteristics, disease self-management experience levels, and users’ values and beliefs [27].

This study also found barriers of patient app usage, for example, a lack of motivation to use the app. This result is in agreement with the findings of a systematic review on engagement with remote measurement technology for managing health, in that participants will find using the app burdensome and may not be motivated to use the app over time [41]. Providers who regularly advocate use of the app would be good motivators. In addition, both theoretical frameworks [30] and empirical studies agree that the perceived interactivity of the app has been the essential predictor of app engagement [42]. As a form of a very important interactivity, healthcare providers’ feedback based on the logged data would be useful to engage and motivate users to use the app. Some studies demonstrate that even health providers’ recommendations to use the app would help motivate the users [43].

### Study barriers

The study took longer than we expected because of several reasons. First, because of the lockdown of COVID-19 pandemic, it took longer to build the app. Second, because of COVID-19 and the opportunity of telehealth, it was difficult to recruit patients in-person in an ambulatory health care setting. Third, if patients who consented encountered technical difficulty during app installation or had app usage questions, they were less likely to get face-to-face help because of the pandemic. Fourth, healthcare providers were often exposed to much longer shifts to meet the demand of health care due to COVID-19, which has caused physical and psychological impacts on healthcare professionals [44]. Therefore, it was very difficult to recruit healthcare professionals who had the time and energy to participate in our study.

### Limitations and future study

Because of the pilot nature of the study, the healthcare providers and the patient app users were limited to the Ohio State University Medical Center. In the future, we plan to test the app usability and feasibility among a more diverse sample, after the app is upgraded, by recruiting participants from other healthcare providers, or recruiting participants online nationally. Second, patients were not directly involved in the app design and development process. In the future, we will update the app by incorporating the feedback (such as patients’ usability and usefulness feedback after 1 month of app use) included in the results of this pilot study to make it more patient centered by this process. Third, in the app, the assessment tool or questionnaire used to detect muscle-related symptoms that are due to satins was not tested for specificity and sensitivity. We will take this into consideration when updating the app in future. Fourth, to simplify the development process, the current app version is only for Apple smartphone users, but future iterations could be more inclusive of users of other smartphone platforms (e.g., Android). In another round of improvement, we anticipate app development that is compatible with various platforms and includes the features to make the app more inclusive across levels of technical literacy (e.g., users with haptic or visual difficulties). Fifth, there could be bias included in the patients’ feedback about the app because patient participants were recruited from one of the authors’ patients, although a verbal statement (i.e., their opinions about the app would not affect their relationship with their healthcare providers) was provided to the participants during the study. This bias could be eliminated in future studies when patient participants will be recruited from other sources that are not related to any of the authors. Sixth, the healthcare providers did not download and use the app and their feedback of the app was based solely on screenshots of the app. This situation occurred for two reasons: 1) healthcare providers were too busy to spend time using the app, especially during the COVID-19 pandemic; and 2) given the resource limitations, only patient participants were allowed to download and use the app. In the future, we plan to provide healthcare providers the access to the upgraded app, and if they have time, they will be allowed to use the app for an extended period before providing their feedback.

## Supplementary Information

**Additional file 1: Appendix 1.** Questions asked in symptom tracking feature of statinterface.

## Data Availability

The datasets generated and/or analyzed during the current study are not publicly available due to participants of this study did not agree for their data to be shared publicly but are available from the corresponding author on reasonable request.
