# Supplementary material for: A mobile health application for patients eligible for statin therapy: app development and qualitative feedback on design and usability
Source: BMC Med Inform Decis Mak. 2023 Jul 19;23:128. doi: 10.1186/s12911-023-02221-4 (PMC10357764; doi:10.1186/s12911-023-02221-4)
Supplement: Supplementary file 1 — Additional file 1: Appendix 1. Questions asked in symptom tracking feature of statinterface. [file 12911_2023_2221_MOESM1_ESM.docx]

Appendix 1 Questions Asked in Symptom Tracking Feature of Statinterface

1. Pain: One a scale from 0 to 10 (0 means no pain at all and 10 means worst pain possible), how do you rate your pain level (when it was at its worst today)?
2. Symptom type:

Please choose from the following that best describe your symptoms (please check all that apply)

Muscle ache Weakness Soreness Stiffness Cramping Tenderness Painful fatigue Burning Stabbing Joint Instability (“giving out”)

Tingling Twitching Shooting Pain Cramps at Night/Before Sleep

Joint Pain Other: please briefly describe: __________________

1. Symptom area (please check all that apply).

Face/Head: Right Left Right more than Left Left more than Right Both equally

Neck: Right Left Right more than Left Left more than Right Both equally

Upper back: Right Left Right more than Left Left more than Right Both equally

Lower back: Right Left Right more than Left Left more than Right Both equally

Hips: Right Left Right more than Left Left more than Right Both equally

Thighs: Right Left Right more than Left Left more than Right Both equally

Knees: Right Left Right more than Left Left more than Right Both equally

Calf/calves: Right Left Right more than Left Left more than Right Both equally

Ankles: Right Left Right more than Left Left more than Right Both equally

Feet: Right Left Right more than Left Left more than Right Both equally

Toes: Right Left Right more than Left Left more than Right Both equally

Shoulders: Right Left Right more than Left Left more than Right Both equally

Arms: Right Left Right more than Left Left more than Right Both equally

Forearms: Right Left Right more than Left Left more than Right Both equally

Hands: Right Left Right more than Left Left more than Right Both equally

My pain is all over or not specific to any area (“generalized”)

1. Symptom start date: When did the pain symptoms start? (please select month and year from the drop down menu)
2. Activities: Have you done anything recently (within prior 7 days) outside your normal routine that may cause muscle pain? (e.g., moved furniture, performed a new or increased intensity physical activity or workout, started a new medication, changed your eating habits)
3. Medication timing: How many medications did you take on time today? (All, Some, None)
4. Skipped medications: List skipped medications (in the text box provided below).

e.g., Atorvastatin - Forgot

1. Health satisfaction: I am satisfied with my overall health.

On a scale from 1 to 10 (1 means strongly disagree and 10 means strongly agree), please indicate your agreement with this statement.
